# Merging statewide data in a public/university collaboration to address opioid use disorder and overdose

**DOI:** 10.1186/s13722-020-00211-9

**Published:** 2021-01-04

**Authors:** William C. Becker, Robert Heimer, Catherine M. Dormitzer, Molly Doernberg, Gail D’Onofrio, Lauretta E. Grau, Kathryn Hawk, Hsiu-Ju Lin, Alex M. Secora, David A. Fiellin

**Affiliations:** 1 Yale School of Medicine, 333 Cedar Street, New Haven, CT 06510 USA; 2 VA Connecticut Healthcare System, 950 Campbell Avenue, Mail Stop 151B, West Haven, CT 06516 USA; 3 Yale School of Public Health, 60 College Street, New Haven, CT 06510 USA; 4 U.S. Food and Drug Administration, 10903 New Hampshire Avenue, Silver Spring, MD 20993 USA; 5 University of Connecticut School of Social Work, 38 Prospect Street, Hartford, CT 06103 USA

**Keywords:** Opioid overdose, Surveillance, Informatics, Opioid use disorder

## Abstract

**Objective:**

Describe methods to compile a unified database from disparate state agency datasets linking person-level data on controlled substance prescribing, overdose, and treatment for opioid use disorder in Connecticut.

**Methods:**

A multidisciplinary team of university, state and federal agency experts planned steps to build the data analytic system: stakeholder engagement, articulation of metrics, funding to establish the system, determination of needed data, accessing data and merging, and matching patient-level data.

**Results:**

Stakeholder meetings occurred over a 6-month period driving selection of metrics and funding was obtained through a grant from the Food and Drug Administration. Through multi-stakeholder collaborations and memoranda of understanding, we identified relevant data sources, merged them and matched individuals across the merged dataset. The dataset contains information on sociodemographics, treatments and outcomes. Step-by-step processes are presented for dissemination.

**Conclusions:**

Creation of a unified database linking multiple sources in a timely and ongoing fashion may assist other states to monitor the public health impact of controlled substances, identify and implement interventions, and assess their effectiveness.

## Background

Connecticut has experienced a fivefold increase in its annual opioid overdose rate over the past decade; it had the 10th-highest overdose death rate among the 50 states in 2018 [1]. In 2018, the number of Connecticut’s opioid-involved fatalities exceeded 1000, a higher total than the next two most common causes of unnatural deaths—motor vehicle accidents and homicide—combined. Connecticut’s overdose crisis has become a major focus of public and political discourse and, increasingly, action.

To help inform the state’s actions, in May 2016 the Connecticut Governor requested creation of a strategic initiative from a team of experts from Yale University’s Schools of Medicine and Public Health [2]. The subsequently named Connecticut Opioid REsponse (CORE) team identified evidence-based strategies that would most effectively and rapidly achieve the overarching mission: to decrease the adverse impact of opioids on Connecticut residents, with an immediate emphasis on reducing overdose mortality. After a three-month effort that included a review of regional and national data, scientific literature, and consultation with stakeholders, six strategies emerged, including one that is the subject of this report—increasing data sharing across agencies and organizations. This approach would merge Connecticut’s key electronic health and addiction treatment center databases to aid timely tracking of progress in achieving the overarching mission.

In this report, we describe the methods used to produce a unified database linking data from multiple sources in a timely and ongoing fashion. Our effort is similar to the Massachusetts Chapter 55 of the Acts of 2015 [3], in that we seek to create a deidentified dataset with person-level records of non-fatal and fatal overdose victims through the merging of multiple state record systems. However, in contrast to the Massachusetts experience, our effort was not state legislature mandated; also, we seek to create a system that will function prospectively, rather than a retrospective cross section of three years’ time. The ultimate goal is a statewide data analytical system that can monitor the public health impact of the opioid crisis in Connecticut, thus allowing for the identification and implementation of near-real-time targeted interventions, evaluation of the efficacy of these interventions and sharing findings among key stakeholders.

## Methods

Based on related work in Maryland [4] adapted to our local context, the CORE team proceeded through six steps to build the data analytic system: (1) stakeholder engagement, (2) articulation of key metrics, (3) funding to establish the system, (4) determination of the data sources needed to track metrics, (5) accessing data and (6) mergingand matching identified patient-level data across the datasets. The initial CORE report can be found here: https://www.ct.gov/dmhas/lib/dmhas/publications/core_initiative10.6.16.pdf.

### Stakeholder engagement

To succeed in the data sharing goal, stakeholders were selected for their access to and knowledge of important datasets and held the necessary authority or influence to facilitate signing of memoranda of understanding (MOUs) to enable cross-agency data sharing agreements. We also identified non-governmental and community-based organizations that influence policy and practice in responding to the increasing rates of overdose. Once the principal governmental and community-based organizations were identified, key individuals within each entity were contacted and invited to participate in meetings designed to build engagement and foster collaboration.

### Articulation of key metrics

With iterative input from stakeholders, the CORE team developed a list of key metrics related to non-fatal and fatal overdose to track progress on the goals of the CORE initiative. Additionally, the team sought to identify data sources and identifiers needed to generate the metrics as described below.

### Funding

Researchers from Yale University (in collaboration with the Mayo Clinic) were awarded funding to establish a Center of Excellence in Regulatory Science and Innovation (CERSI) by the Food and Drug Administration (FDA). The overall goals of CERSI are to create infrastructure for regulatory science knowledge generation, conduct research to address key gaps in knowledge, and develop tools to support regulatory decision-making and the overall mission of FDA. The CORE team received CERSI project funds to support the development work described in this report.

### Identifying data elements and data sources

**Fig. 1:**
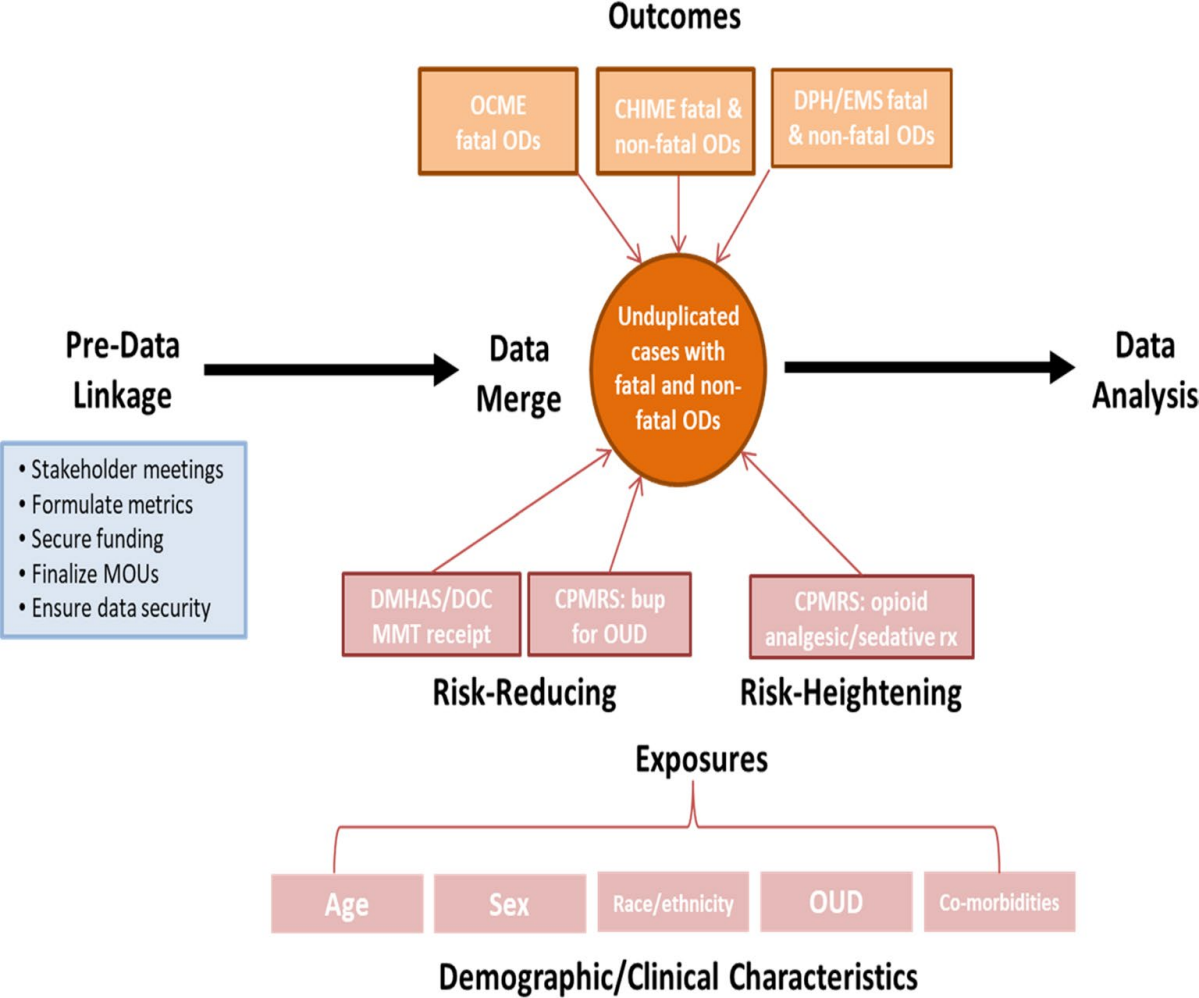
Model of hypothesized relationships between exposures and outcomes based on published research

To guide the project, the CORE team considered established relationships among prescription opioid and other controlled substance *exposures*, defined as factors predisposing (e.g., increased milligram morphine equivalent doses) or reducing (e.g., buprenorphine for medication-assisted treatment) the risk of opioid overdose, and *outcomes*, defined as non-fatal or fatal overdoses (see Fig. 1).

Once exposures and outcomes were defined, state agencies and institutions were queried to determine if they collected relevant data with sufficient detail to match individuals across datasets from different state agencies. We also ascertained their protocols and procedures with respect to sharing data. To aid long-term sustainability, we decided to have the merged data remain within state agencies and universities rather than have them transferred to Yale University or another outside entity. Since the University of Connecticut (UConn) had a contract with the Department of Mental Health and Addiction Services (DMHAS) and already had access to some of the datasets it was determined that UConn would host the data.

### Accessing data

Each state agency needed to ensure that regulatory and policy restrictions limiting the use of personal identifiers would not be violated, including privacy laws, Health Insurance Portability and Accountability Act (HIPAA) requirements, and human subjects review. All agencies were assured that, prior to conducting analyses, identifiers would be removed from the dataset (with some being replaced by higher-level groupings, e.g., 3-digit zip codes replacing full 5-digit zip codes), and that the analytic dataset would be available for use by the state agencies.

To preserve confidentiality, maximize efficiency and minimize potential or perceived undue influence from external partners, the individual and merged data resides within Connecticut state agencies. Decisions about how the data is analyzed are dictated by funding (e.g. FDA) and internal requests (e.g. Connecticut Department of Mental Health and Addiction Services) in collaboration with CORE colleagues.

### Data merging and matching

To explore associations between the exposures and outcomes noted above, we planned to merge datasets and match individual identifiers to create a per-individual-subject dataset for analysis. The step-by-step details of that process, including how data are analyzed and stored within the state, are listed below.

## Results

### Stakeholder engagement

Stakeholder meetings were conducted from June through November 2016 to obtain input on all components of the strategic initiative, including the data sharing strategy (for full list of entities see Additional file 1: Appendix). We benefitted from the experience these entities had accrued in their established efforts to address the opioid crisis. Stakeholders in attendance included a range of state and local government, non-government, and commercial actors as well as patient and harm reduction advocacy groups, and civil society partnerships (such as the Governor’s Alcohol and Drug Policy Council). We began each stakeholder meeting by affirming our intent to be open and collaborative, seeking input on our aims and methods, and repeatedly highlighting the shared mission of all involved. Follow-up meetings to continue to encourage completion of MOUs were held over the ensuing several months.

### Articulation of key metrics

Based on iterative stakeholder feedback, the final key metrics included (1) the proportion of individuals experiencing fatal and non-fatal overdoses who received controlled substance prescriptions reported in the Connecticut Prescription Monitoring and Reporting System (CPMRS); (2) the timing of those prescriptions relative to an overdose event; (3) the proportion of individuals receiving opioid agonist treatment with methadone or buprenorphine or engaging with an abstinence-based treatment program at the time of overdose; (4) the timing between withdrawal from different treatments for opioid use disorder (OUD) and overdose events; and (5) the proportion of overdose survivors who become linked to opioid agonist treatment within 90 days of the overdose event. With these metrics, we sought to determine (1) the extent to which the CPMRS did or did not identify a large proportion of individuals at risk for overdose due to prescribing profiles (e.g. high opioid dose, opioids in combination with benzodiazepines) (Metrics 1 and 2); (2) the impact of opioid agonist treatment on decreasing rates of overdose (Metric 3); (3) the impact of withdrawal from treatment on overdose (Metric 4); and (4) the extent to which overdose survivors were linked to treatments known to decrease overdose mortality (Metric 5).

### Funding

The CORE team’s proposal to the Yale-Mayo Clinic CERSI was funded by the FDA after three rounds of feedback on the proposed methods. In addition, recognizing a need for expertise in state databases, the Yale University group invited colleagues at University of Connecticut to collaborate on the project.

### Identifying data sources

With respect to exposures, we distinguished between those that heighten overdose risk, with a focus on published relationships between opioid analgesic dose [5], concomitant opioid and benzodiazepine prescriptions [6], recent release from incarceration [7], or cessation of treatment for OUD [8], and those that may reduce overdose risk, such as use of sublingual buprenorphine products and methadone for the treatment of OUD [9]. In Connecticut, controlled substance prescription data (including buprenorphine products) is collected and managed by the Department of Consumer Protection (DCP). Data related to methadone treatment in the state is managed by the Department of Mental Health and Addiction Services (DMHAS) (see Table 1).

**Table 1:** Data sources relevant to opioid-related overdoses

	Data elements	Dataset	Stakeholder
Exposures	Controlled substance prescriptions (opioid analgesics, methadone, and benzodiazepines)	Connecticut Prescription Monitoring and Reporting System (CPMRS)	Department of Consumer Protection
	Buprenorphine for opioid use disorder (OUD)	CPMRS	Department of Consumer Protection
	OUD treatment, including methadone from opiate treatment programs		Department of Mental Health and Addiction Services
	Incarceration periods, methadone for OUD		Department of Correction
Outcomes	Fatal opioid overdoses		Office of the Chief Medical Examiner
	Fatal and nonfatal opioid overdoses	ChimeData	Connecticut Hospital Association
	Out of hospital opioid overdoses		Department of Public Health/Department of Emergency Services and Public Protection

With respect to outcomes, we focused on the collection of data on both fatal and non-fatal cases. Compared to data pertaining to non-fatal cases, fatal overdose information is typically more accurate because of the involvement of the Office of the Chief Medical Examiner (OCME), which performs an autopsy and toxicological testing when indicated in all unnatural deaths. Because of the detailed fatal case reports, we differentiated between single-, poly-opioid, and poly-substance related events, and whether the overdose was intentional or unintentional.

For non-fatal overdoses, the source of information and level of detail depends on where the victim is encountered. Overdose reversals with naloxone are often performed by first responders, which can include emergency medical service (EMS) personnel, local police and firefighters, and, in the cases of many of the smaller Connecticut towns, the state police. Uniformly detailed reporting on these cases is not mandatory. The Department of Public Health (DPH) maintains the database for EMS reversals collected through National EMS Information Systems forms. The Department of Emergency Services and Public Protection (DESPP) collects data from the state police. Reversals can also be performed by laypeople in the community, but there is very little reliable information on naloxone administration unless individuals performing the reversal engage with the EMS or healthcare systems at the time of the event or subsequently report it to a community program that supplied the naloxone. The Connecticut Hospital Association’s ChimeData collects and maintains administrative discharge (UB-04 claims-based) data from inpatient admissions, hospital-based outpatient surgery, and emergency department (ED) non-admissions and provides this to the DPH.

### Accessing data

Of these datasets, the one most readily obtained was from the OCME, aided by the fact that two members of the CORE team (LEG, RH) have had long-standing relationships with the OCME working on similar projects [10]. The CORE team reviews autopsy reporting for each opioid-involved fatality to determine if it was unintentional or intentional and if the final immediate cause of death involved an opioid (± other substances) as determined by either the medical examiner and/or toxicological evidence. Research involving data on deceased individuals is IRB-exempt and some information is publicly available, but an agreement had to be established between the OCME and the research team as most of the detailed decedent-level data are not publicly available. Department of Correction (DOC) data are publicly available, though, similar to OCME data, specific elements of most interest to our work (e.g. receipt of methadone during incarceration) are not.

In contrast, almost all other data sought by the CORE team are considered protected personal health information, and as a consequence, the agencies that maintain the databases have identified HIPAA and other barriers that create legal challenges to sharing data within state agencies and with the CORE team. Overall, developing a satisfactory protocol and obtaining the approvals required over a year’s time and review by multiple attorneys. A key factor in our eventual success was that identifiers were only linked between state agencies by a designated member of our team, a UConn researcher who had the credentials required to view confidential data from two of the state agencies and who worked closely with other agency employees to conduct the linkage in a manner consistent with state protocols for data security.

### Data merging and matching

We used a public domain software program that integrates both probabilistic and deterministic matching algorithms (The Link King V9, www.the-link-king.com) to identify and match individual records across multiple agencies using all available demographic identifiers and geographic information, such as residential address and zip code [11]. The deterministic protocol ascertains whether record pairs matched or did not match on a set of established criteria; the Link King application employs a complex deterministic protocol that allows some discrepancy on the record elements through “fuzzy” equivalence algorithms. It also includes an array of probabilistic algorithms such as phonetic name matching, approximate string matching and spelling distance, and calculation of distance between the geographic centers of zip codes. These probabilistic procedures use statistical formulae to calculate an overall similarity score between data elements for each record pair and cut points to determine if the records were from the same individual. In a study using similar data elements, the Link King application was shown to have high accuracy for records linkage, with sensitivity at 96.6% and positive predictive at 96.1% [12].

The record matching was carried out in two phases. The first phase involved linking records across data extracts from administrative databases concerning hospital care, emergency medical treatment, medical examiner reports, incarceration dates, and substance use disorder treatment through the state secured data exchange protocol. A randomly generated unique identifier was assigned to each matched individual. Once datasets were linked through the unique identifier, all other personal identifiers were removed from these datasets, except the master individual list file. In the second phase, the master file was transported via a state securely encrypted laptop, to the state agency—the DCP—that administers the state’s PDMP. Because the DCP does not allow identified data to leave its premises, the record matching was performed on site and all identifiers were removed prior to departure. After the second phase matching was completed, individual identifiers were stripped and only the unique identifier and de-identified data were saved in the laptop to be merged with the de-identified data extracts from the first phase.

As above, we identified both exposure and outcome datasets with personal identifiers—including sex, age, race/ethnicity and census tract allowing for inference of socioeconomic status—with sufficient detail for project goals. For exposure ascertainment, we included CPMRS and DMHAS treatment datasets, DOC data, ChimeData, and, for outcome ascertainment, we included OCME cause-of-death files (fatal opioid-related events), ChimeData (fatal and non-fatal overdose events), EMS and DESPP data (non-fatal overdose events).

With exposure and outcome data matched, we generated a per-subject profile of the following: (1) prescription opioid and benzodiazepine receipt including products, dosages, duration of therapy, and timing in relation to outcome; (2) hospitalizations including duration, admitting and discharge diagnoses, controlled substances received, and timing in relation to outcome; (3) DMHAS- and DCP-captured OUD treatment episodes, categorized as detoxification episodes, longer-term abstinence-based treatment, and methadone and buprenorphine treatment episodes, and (4) DOC episodes including arrest charge, duration of incarceration, methadone treatment, and timing in relation to outcome. For DMHAS exposure data, elements included OUD diagnoses, OUD medication treatment received, start and end dates of treatment, and timing in relation to outcomes in the cohort.

## Discussion

In response to the public health crisis of opioid overdose in Connecticut, the CORE team was created to identify ways to decrease the adverse impact of opioids on Connecticut residents. One of the six strategies that emerged was to link and merge key electronic health and social service databases to aid timely tracking of progress in achieving the overall mission [13]. As valid measurement is critical to assessing impact of policy changes and other public health interventions, herein we described the methods for creating a person-level dataset, including all the component steps in the process, that will ultimately be used for real-time analysis. While other reports have described important state-level efforts to curb overdose rates [14, 15] and the steps required for database linking in Maryland [4], this report adds to the literature by describing a more recent process for database linking and providing detailed instructions formatching individual records across datasets. Ultimately, per-subject profiles will be analyzed individually and in aggregate using descriptive, bivariate, and multivariable methods to test for associations between exposures and outcomes. The availability of person-level sociodemographic information will improve understanding of differential impacts of the opioid crisis on vulnerable subgroups and potential disparities in treatment access.

Our work was facilitated by a variety of factors. First, the CORE project was initiated, and initially promoted, by the Governor’s office, which provided early momentum to unite stakeholders in a common mission. As we did, readers may wish to consult the National Governors Association report on developing stakeholder consensus and promoting buy-in related to addressing the opioid crisis [16]. Second, among Yale University, University of Connecticut, and FDA collaborators, we had strong subject matter expertise and experience with state database management and epidemiologic analyses. Third, funding by the FDA through the Yale-Mayo Clinic CERSI protected time for researchers to devote to the project. For researchers in other states who may not have federal grant funds, seeking local private foundation grant opportunities where the health of the state’s citizens is the funder priority or negotiating contracted funding from the state’s Department of Health are options to consider.

However, we encountered barriers to project success. Momentum to execute the project was greatest at inception, when the Governor first named the CORE team. At that point, levels of motivation from the Governor’s office, state agencies, and the CORE team were high and goals were aligned. As months went by, the CORE team’s motivation remained high but delays in obtaining data and MOUs meant that the priorities of state agencies had changed to other opioid-related projects. Without a legislative mandate for coordinated effort across state agencies, our team needed to establish several different MOUs that each required a lengthy internal state agency legal review. With each agency following its own legally-mandated process, our ability to track a dynamic public health crisis was significantly slowed. Strategies such as those undertaken in Massachusetts whereby the processes for linking datasets was part of legislation (namely, Chapter 55), may have yielded a more expedited process. Sustainability of the Connecticut project will likely depend on legislative mandates, demonstrated utility and rechanneled funding.

Our methods have limitations. Chiefly, the steps we took were adapted to the local Connecticut context and may not generalize to all states. Second, the key metrics we chose excluded—because of time and funding constraints and funder priorities—other important metrics such as naloxone distribution that may be of higher priority in other states.

## Conclusion

The methods listed in this report detailing creation of a unified database linking multiple sources in a timely and ongoing fashion may assist other states to monitor the public health impact of controlled substances, identify and implement interventions, and assess their effectiveness.

## Supplementary Information

**Additional file 1: Appendix.** Entities engaged in stakeholder meetings June through November 2016 to obtain input on the data sharing strategy

## Data Availability

The datasets generated and/or analyzed during the current study are not publicly available due to the sensitive nature and personal identifiers.
