# Supplementary material for: Merging statewide data in a public/university collaboration to address opioid use disorder and overdose
Source: Addict Sci Clin Pract. 2021 Jan 4;16:1. doi: 10.1186/s13722-020-00211-9 (PMC7780404; doi:10.1186/s13722-020-00211-9)
Supplement: Supplementary file 1 — Additional file 1: Appendix. Entities engaged in stakeholder meetings June through November 2016 to obtain input on the data sharing strategy [file 13722_2020_211_MOESM1_ESM.docx]

**Additional File 1: Appendix**

Entities engaged in stakeholder meetings June through November 2016 to obtain input on the data sharing strategy:

**Connecticut state agencies, legislatively mandated entities, and elected officials**

Alcohol and Drug Policy Council

Department of Mental Health and Addiction Services

Department of Children and Families

Department of Consumer Protection

Department of Corrections

Department of Emergency Services and Public Protection

Department of Public Health

Department of Social Services

Insurance Department

Office of the Chief Medical Examiner

Office of the Governor

Office of Policy and Management

Southeastern Mental Health Authority

State Representative Theresa Conroy

State Senator Terry Gerratana

**City agencies**

Hartford, Department of Health and Human Services

New Haven, Health Department and Community Services

Administration

New London, Office of Human Services

Waterbury, Department of Health

**Federal agencies**

Office of the National Drug Control Policy, New England High Intensity Drug Trafficking Area

United States Department of Veterans Affairs, VA Connecticut Healthcare System

**Non-governmental agencies**

Access Health CT

AIDS Project Connecticut

AIDS Project Greater Danbury

Alliance for Living

American Academy of Addiction Psychiatry, Provider’s Clinical Support System for Medication Assisted Treatment

American Society of Addiction Medicine, Connecticut Chapter

APT Foundation

Beacon Health Options

Charlotte Hungerford Hospital

Community Health Resources

Connecticut Association of Addiction Professionals

Connecticut Community Nonprofit Alliance

Connecticut Counseling Centers

Connecticut Hospital Association Emergency Department Directors Meeting Group

Connecticut Prevention Network

Greater Hartford Harm Reduction Coalition

Hartford Dispensary

InterCommunity

Liberation Programs

Litchfield County Opiate Task Force

Multicultural Ambulatory Addiction Services (MAAS)

Newtown Prevention Council

Optimus Health Care

Recovery Network of Programs

Rushford

University of Connecticut Health Center

University of Connecticut School of Medicine

Yale University School of Medicine

Yale University School of Public Health
